# Determination of essential oil and biological activities of *Hypericum ternatum* Poulter and *H. scabrum* L. species collected from different localities: is *H. scabrum* an alternative to multifunctional species ST JOHN’S WORT (*H. perforatum*)?

**DOI:** 10.55730/1300-0527.3494

**Published:** 2022-08-13

**Authors:** Mehmet AKDENİZ, İsmail YENER, Sevgi İRTEGÜN KANDEMİR, Şafak ÖZHAN KOCAKAYA, Mehmet FIRAT, Serkan YİĞİTKAN, Nesrin HAŞİMİ, Abdulselam ERTAŞ, Ufuk KOLAK

**Affiliations:** 1 The Council of Forensic Medicine, Diyarbakır Group Chairmanship, Diyarbakır, Turkey; 2 Department of Analytical Chemistry, Faculty of Pharmacy, Dicle University, Diyarbakır, Turkey; 3 Department of Medical Biology, Faculty of Medicine, Dicle University, Diyarbakır, Turkey; 4 Department of Organic Chemistry, Faculty of Science, Dicle University, Diyarbakır, Turkey; 5 Department of Biology, Faculty of Education, Van Yüzüncü Yıl University, Van, Turkey; 6 Department of Pharmaceutical Botany, Faculty of Pharmacy, Dicle University, Diyarbakır, Turkey; 7 Department of Biology, Faculty of Science, Batman University, Batman, Turkey; 8 Department of General and Analytical Chemistry, Faculty of Pharmacy, İstanbul University, İstanbul, Turkey

**Keywords:** *Hypericum ternatum*, *Hypericum scabrum*, essential oils, anticholinesterase, antimicrobial, molecular docking

## Abstract

The importance of *Hypericum* L. species, being used in traditional medicine, in the scientific world is increasing day by day. *Hypericum* species are plants that have been used in the treatment of many diseases for a long time and have healing properties. In the current study, the essential oil compositions of *Hypericum scabrum* L. and *H. ternatum* Poulter collected from different localities in Turkey were determined by GC-MS/FID. In addition, their antioxidant, cytotoxic, and antimicrobial activities with their enzyme inhibitory potentials (cholinesterase, urease, tyrosinase, elastase, and collagenase) were investigated. Also, in vitro and in silico studies of the major components of the species have been carried out on the studied enzymes. It was determined that four *H. scabrum* samples mainly contained α-pinene (55.99%–62.80%) while three *H. ternatum* samples contained 2-methyloctane (9.45%–22.39%) and α-pinene (12.75%–33.08%). While *H. scabrum* essential oils possessed significant biological activity potential except for the antimicrobial activity, *H. ternatum* samples did not. All essential oil samples of *H. scabrum* exhibited a high cytotoxic effect (IC_50_ 21.67 ± 0.34 to 34.67 ± 0.45 μg/mL) against colon cancer cell line (HT-29) and indicated higher acetyl-(Inhibiton% 83.30 ± 1.90 to 93.08 ± 1.04) and butyryl-cholinesterase (Inhibiton% 80.58 ± 1.19 to 93.28 ± 1.99) inhibitory activity than the standard compound, galantamine. Furthermore, all samples of *H. scabrum* showed high tyrosinase (Inhibiton% 69.00 ± 1.64 to 95.25 ± 1.42) and elastase (Inhibiton% 27.58 ± 0.34 to 36.69 ± 0.18) inhibitory effects. These biological activity results indicated that *H. scabrum* essential oil could be used in the cosmetic and pharmaceutical industries.

## 1. Introduction

The genus *Hypericum* L. belongs to the family Hypericaceae and there are approximately 500 species in the world. Turkey is an important center for the genus *Hypericum* where 48 of 97 species are endemic [1]. *Hypericum* species, especially *H. perforatum* L. (St. John’s wort), have been used externally (wounds, inflammation of the skin, etc.) and internally (antidepressant) in the treatment of many diseases since ancient times [2]. More than 3000 studies have been published about this genus, primarily *H. perforatum* in the last decade [3]. Many investigations on the *Hypericum* species have been carried out to demonstrate their various biological activities such as tyrosinase, collagenase, elastase, and hyaluronidase inhibitory activities, as well as antiinflammatory, wound healing, and antimicrobial activities related to the cosmetic field [3]. In addition, there are also plenty of studies about the neuropharmacological potential of this genus [4].

There are many works in the literature regarding the essential oil contents of *Hypericum* species. Hydrocarbons such as 2-methyloctane, nonane, and undecane, monoterpenes such as α-pinene, limonene, **β**-myrcene and cis-β-ocimene, and sesquiterpenes, particularly caryophyllene and caryophyllene oxide are predominant compounds in their essential oil contents. Previous studies reported that α-pinene (45.6%), limonene (18.52%), and β-pinene (11.64%) for *H. scabrum* L. [5]; caryophyllene oxide (24.33%), α-pinene (14.82%), and caryophyllene (11.41%) for *H. lysimachioides* Boiss var. *spathulatum* Rabson [6]; germacrene-D (21.7%), β-caryophyllene (18.3%), and δ-cadinene (6.4%) for *H. triquetrifolium* Turra. [7] were identified as their major compounds. Similar studies reported also that α-pinene (31.9 **±** 1.9%), (E)-β-ocimene (12.5 **±** 1.0%), β-phellandrene (8.4 **±** 1.1%), and β-pinene (6.3 **±** 0.4%) for *H. helianthemoides* (Spech) Boiss [8]; α-pinene (19.0%), germacrene D (12.5%), and β-pinene (8.7%) for *H. empetrifolium* Willd [9]; β-selinene (15%), caryophyllene oxide (9%), β-caryophyllene (8%), and **γ**-muurolene (7%) for *H. pruinatum* Boiss [10]; α-pinene (57.3%), β-pinene (9.0%), and limonene (6.2%) for *H. hyssopifolium* Chaix. var. *elongatum* (Ledeb) Woron [11]; *tr*-caryophyllene (23.92%), limonene (11.19%), and α-cadinol (10.67%), caryophyllene oxide for *H. pseudolaeve* Robson [12]; α-pinene (71.2%), caryophyllene oxide (2.9%), and caryophylladienol I (0.6%) for *H. lydium* [13]; and germacrene D (30.2%) and α-pinene (21.5%) for *H. confertum* Choisy [14] were determined to be the predominant compounds in the essential oils.

While there are no studies on *H. ternatum* Poulter in the literature, there are many studies on *H. scabrum* [4,5,8,15,16]. This is the first study on the phytochemical and biological activity of *H. ternatum* essential oil, and also that on the cytotoxic, anticholinesterase, antiurease, antityrosinase, antielastase, and anticollagenase activities of *H. scabrum* essential oil.

The aim of the present study was to determine the essential oil compositions of *H. scabrum* and *H. ternatum* collected from four and three regions, respectively, in Turkey. Their antioxidant (DPPH free radical and ABTS cation radical scavenging activity, and CUPRAC methods), anticholinesterase, antiurease, antityrosinase, antielastase, and anticollagenase potentials were also investigated. In addition, the toxic effects of the essential oils on primary dermal fibroblast (PDF) and their cytotoxic effects on MCF-7 (breast) and HT-29 (colon) cancer cell lines were investigated with the MTT method. Moreover, the disc broth dilution method and minimum inhibitory concentration were used to determine their antimicrobial activity. Both in vitro and in silico enzyme studies of the major components, which were detected at a high rate according to the GC-MS results of all samples, were carried out to reveal the content–biological activity relationship.

## 2. Materials and methods

### 2.1. Plant material

*Hypericum scabrum* L. (S-1) was collected in May 2015 by Dr. Abdulselam Ertaş from Nevşehir/Cappadocia and identified by Dr. Yeter Yeşil (İstanbul University Faculty of Pharmacy, Deparment of Pharmaceutical Botany). *H. scabrum* (S-2) from Diyarbakır/Elaziğ Road, *H. scabrum* (S-3) from Kahramanmaraş/Ahır Mountain, *H. scabrum* (S-4) from Van/Bahçesaray/ Yukarı Nalıca, *H. ternatum* Poulter (S-5) from Amasya/İlyas Village, *H. ternatum* (S-6) from Tekirdağ/Malkara, and *H. ternatum* (S-7) from Afyonkarahisar/Şuhut were collected in July 2016 and identified by Mehmet Fırat (Van Yüzüncü Yıl University, Faculty of Education, Department of Biology). Voucher specimens were deposited in the Herbariums of İstanbul University (ISTE) and Van Yüzüncü Yıl University Faculty of Science (VANF). Herbarium numbers of S1–7 samples are ISTE 113 611, M. Firat 32873 (VANF), M. Firat 32904 (VANF), M. Firat 32921 (VANF), M. Firat 32889 (VANF), M. Firat 32908 (VANF), and M. Firat 32915 (VANF), respectively.

### 2.2. GC-MS analyses

In this study, the essential oil contents of the samples were analyzed using Agilent brand 7890A Model GC/FID gas chromatography and Agilent brand 5977B model mass spectrometer (MS). Components of essential oils (shadow-dried aerial parts) obtained by hydrodistillation method using Clevenger apparatus were determined by GC-MS/FID [6,17]. HP-5MS UI capillary column (30 m to 0.25 mm and 0.25 μm film thickness) was used. The injector temperature was adjusted to 250 °C. Split flow and split ratios were 25 mL/min and 25:1, respectively. The injection volume was 1.0 μL. Mass spectra were detected at 70 eV and the mass range was m/z 40–500 amu. GC oven temperature started at 50 °C and held at this temperature for 4 min and then ramped to 240 °C at a rate of 3 °C per minute and held at the final temperature for 5 min. Helium was used as a carrier gas at a flow rate of 1 mL/min. The MSD and FID detector’s temperatures were 230 °C and 300°C, respectively. Data were collected from both MS and FID detectors at the same time with the help of a separator installed at the exit of the column. While qualitative identification of the components was completed with the MS data, quantitative and percentage results were made with the data collected from the FID detector. For this reason, all parameters except for the temperatures of MS and FID detectors were the same.

Alkanes (C7 – C40) were used as reference points in the calculation of retention indices (RI) using the same conditions. The compounds were identified by comparing their retention times and mass spectra with those obtained from authentic samples and/or the NIST and Wiley spectra as well as data from the published literature.

### 2.3. Antioxidant activity

ABTS cation radical [18] and DPPH free radical scavenging activity [19], and CUPRAC (copper (II) ion reducing antioxidant capacity) [20] methods were used to determine the antioxidant properties of the samples. In these three antioxidant test methods, α-tocopherol and BHT (butylated hydroxytoluene) were used as standards. IC_50_ calculations were performed by using the samples with 250, 100, 50, 25, 10, and 1 μg/mL concentrations [21,22].

### 2.4. Cytotoxic activity

In order to determine the toxic and cytotoxic effects of the samples, the MTT method developed by Mojarraba et al. [23] was used with minor modifications. The toxic effects of the samples were studied against PDF (healthy primary dermal fibroblast) cell line, while their cytotoxic effects were studied against HT-29 (colon cancer) and MCF-7 (breast cancer) cell lines [24,25]. MTT assay was performed 48 h after treatment. Ten microliters of MTT solution (5 mg/mL) were added to each well, and cells were incubated for 3 h at 37 °C with 5% CO_2_, 95% air, and complete humidity. After 3 h, the medium was removed and replaced with 100 μL of DMSO. The plates were put on a plate shaker at room temperature for 15 min and the optical density (OD) of the wells was determined at a wavelength of 540 nm using a plate reader (Multi Scan Go, Thermo). IC_50_ calculations were performed by using the samples with 250, 100, 50, 25, 10, and 1 μg/mL concentrations.

### 2.5. Anticholinesterase activity

The spectrophotometric method based on acetyl-(AChE: from electric eel, Type-VI-S, Sigma) and butyryl-cholinesterase (BChE: from horse serum, Sigma) inhibitions developed by Ellman et al. [26] was used to determine the anticholinesterase capacity of the samples. Galantamine was used as a standard compound.

In all enzyme inhibition methods, inhibition% values of the samples were calculated at a concentration of 100 μg/mL. Besides, the same volume of ethanol was used in the enzyme inhibition methods as the negative control. Ethanol (99.9%, Merck) was used to prepare the stock solutions and to dilute the solutions in all enzyme experiments.

### 2.6. Antiurease activity

The method developed by Hina et al. [27] was used to determine the urease (from *Canavalia ensiformis*, Type III, Sigma) inhibitory activity of the samples. Thiourea was used as a standard for the urease activity test method.

### 2.7. Antiaging activity

The results of tyrosinase (from *mushroom*, Sigma) elastase (from *Porcine pancreas*, Type I, Sigma), and collagenase (from *Clostridium histolyticum*, Type I, Sigma) inhibitory activities were used to determine the antiaging potentials of the samples.

The method developed by Hearing and Jimenez [28] was used for tyrosinase inhibitory activity with slight modifications, and kojic acid was used as a standard.

Elastase inhibitory activity was determined according to the protocol developed by Kraunsoe et al. [29] with slight modifications. Ten microliters of the sample ethanol solution and 20 μL of elastase enzyme solution were added to 40 μL (0.1 M Tris-Cl, pH = 8) of buffer solution and incubated for 10 min (37 °C). Afterwards, 30 μL of 1.015 mM substrate (N-succinyl-(Ala)-3-nitroanilide) solution which was prepared with buffer solution (0.1 M Tris-Cl, pH = 8) was added and incubated at 37 °C for 20 min. Next, absorbance values were measured at 410 nm. Oleanolic acid was used as a standard.

Collagenase inhibitory activity was determined according to the protocol developed by Thring et al. [30] with slight modifications. Sample solution prepared in 20 μL of DMSO and 10 μL of collagenase enzyme solution (0.8 U/mL) were added to 50 μL of phosphate buffer (pH 7.5) and incubated at 25 °C for 15 min. Afterwards, 20 **μL** of substrate solution (N-(3-[2-Furyl]acryloyl)-Leu-Gly-Pro-Ala) was added, and incubated at 25 °C for 20 min, then absorbance values were measured at 340 nm. Epicatechin gallate was used as a standard compound.

The following equation was used to calculate the AChE, BChE, urease, tyrosinase, elastase, and collagenase enzyme inhibition capacity of the samples.


AChE, BChE, urease, tyrosinase, elastase and collagenase inhibition (%)=100-(OD test well/OD control)×100.

### 2.8. Molecular docking

The compatibility and activities of 2-methyloctane, α-pinene, β-pinene, limonene, and 2-methyloctane compounds, which were determined as major in the species, to the active site of anticholinesterase, antityrosinase, antielastase, and anticollagenase enzymes were determined using the Dock 6.5 program (Kuntz Lab programs are available free of charge for academic institutions) [32]. A molecular modeling study was not performed because none of the samples has antiurease activity. Related in silico studies (AChE, BChE, urease, tyrosinase, elastase and collagenase enzymes) were carried out according to Yener et al. [25]. X-ray pdb models were obtained from Protein Data Bank (2×8b.pdb for AChE, 4bbz.pdb for BChE, 5i38.pdb for tyrosinase, 1bru.pdb for elastase, 2d1n.pdb for collagenase) [31–35]. Crystallographic water molecules were removed from all the structures and the missing coordinates of the atoms were modeled using xLeAP and an ff99SB force field. Atoms on proteins were assigned the PARM99 charges, and all ionizable residues were set at their default protonation states at neutral pH. All structures were further processed by the xLeAP module of AMBER. The molecular systems were neutralized by the addition of counterions. The selected target proteins were minimized with Amber force field [36–40]. The Discovery Studio 4.1 Client, a visualizing tool, was used to generate the hydrophobicity graphs and graphical depiction of target proteins. The protein Ramachandran graph was accessed through PDB. The Discovery Studio 2.1 Client [41] was used to view 3D structure of target proteins.

#### 2.8.1. Docking studies

Dock 6.5 [32] module allows all stages of a docking process to be performed with the generation of ligand conformations, ligand docking, and the scoring of the binding modes. As in this case, where a rigid receptor approximation was used, it is expected that 130 different receptors considered will lead to different ligand binding modes depending on the initial size of the enzyme-binding cavity. Thus, fourteen designed molecules were docked onto available receptors following a multistep procedure. In order to describe receptor-binding properties, a grid of potential energy was calculated for atoms taking part in the binding pocket. These atoms were obtained from the analysis of each protein-ligand complex. In this step, default parameters were used. The ligand was then docked using the calculated grid to place it into the cavity and score the proposed binding mode.

### 2.9. Antimicrobial activity

Antimicrobial activity was determined against gram-negative (*Escherichia coli* ATCC25922, *Pseudomonas aeruginosa* ATCC27853), gram-positive (*Staphylococcus aureus* 25923, *Streptococcus pyogenes* ATCC19615) bacteria and yeast (*Candida albicans* ATCC10231) by disc broth dilution method [42,43] as minimum inhibitory concentration. Ninety-six well plates containing 0.1 mL of Mueller-Hinton broth and 0.1 mL of different concentrations of essential oils (range from 2500 μg/mL to 1.22 μg/mL) were inoculated with 0.005 mL of culture equal to 10^5^ CFU/mL. After incubation at the appropriate temperature and time, MIC values were determined. Ampicillin and fluconazole were used as positive controls for the bacteria and yeast, respectively. All tests were done in triplicate.

### 2.10. Statistical analysis

The results of the activity assays were shown as means ± standard error meaning. The results were evaluated using an unpaired *t*-test and one-way analysis of variance ANOVA. The differences were regarded as statistically significant at p < 0.05.

## 3. Results and discussion

### 3.1. Essential oil content

The essential oil compositions of *H. scabrum* (S-1, S-2, S-3, S-4) from four localities and *H. ternatum* (S-5, S-6, S-7) collected from three places in Turkey were detected by GC-MS/FID (Table 1, Figures 1A and 1B). Their essential oil compositions consisted of 64 compounds that were found to be between 90.09% and 97.70% (Table 1). Major components were determined as α-pinene (56.25%), β-pinene (13.67%), and limonene (8.46%) for S-1; α-pinene (62.8%), β-pinene (11.25%), and β-myrcene (8.06%) for S-2; α-pinene (58.61%), β-pinene (5.11%), and germacrene D (4.10%) for S-3; α-pinene (55.96%), germacrene D (6.42%), and bicyclogermacrene (3.69%) for S-4; 2-methyloctane (18.67%), α-pinene (16.45%), and germacrene D (8.63%) for S-5; α-pinene (33.08%), 2-methyloctane (22.39%), and β-pinene (10.22%) for S-6; α-pinene (12.79%), caryophyllene (9.50%), and 2-methyloctane (9.45%) for S-7. α-Pinene was found to be the major constituent in *H. scabrum* samples collected from Nevşehir (S-1, 56.25%), Diyarbakır (S-2, 62.80%), Kahramanmaraş (S-3, 58.61%), and Van-Bahçesaray (S-4, 55.96%) regions. These results indicated that the essential oil compositions of *H. scabrum* samples were quite similar, and the regional variation caused minor changes in their compositions. The main components of *H. ternatum* samples collected from Amasya (S-5), Tekirdağ (S-6), and Afyon (S-7) regions were determined to be 2-methyloctane (18.67%, 22.39%, and 9.45%, respectively), and α-pinene (16.45%, 33.08%, and 12.79%, respectively). Similarly, the regional differences did not affect the compositions of *H. ternatum* essential oils. As a result, α-pinene was the predominant compound of the studied samples, and all samples were found to be rich in terpenes (Table 1).

While there are several studies in the literature about the essential oil compositions of *H. scabrum*, there is no research on that of *H. ternatum*. Bağcı and Bek**ç**i [5] reported that α-pinene (45.60%), limonene (18.52%), and β-pinene (11.64%) were determined to be the main components in *H. scabrum* essential oil. Another study demonstrated that α-pinene (50.0%), β-pinene (9.7%), and limonene (6.6%) were also found to be major compounds in *H. scabrum* [8]. In other studies, α-pinene was defined to be the main compound that was present at about 50% in the essential oil of the species [15]. Our results on the essential oil contents of *H. scabrum* were in agreement with those of the literature and indicated that the major component and amount (approximately 50% α-pinene) of its essential oil were not related to its growing regions.

### 3.2. Antioxidant and toxic-cytotoxic activities

The antioxidant potentials of the tested *Hypericum* essential oil samples were determined using DPPH free radical and ABTS cation radical scavenging, and CUPRAC assays (Table 2). None of the tested samples showed DPPH free radical scavenging activity. The sample S-1 exhibited the best activity in both ABTS cation radical scavenging (IC_50_ 38.04 ± 0.65 μg/mL) and CUPRAC (A_0.5_ 7.95 ± 0.45 μg/mL) methods among the tested essential oils. In addition, it was found that the S-2 sample (A_0.5_ 10.60 ± 0.65 μg/mL) was less active than the S-1 sample determined by CUPRAC antioxidant activity method, and these two samples were appeared to be more active than α-tocopherol (A_0.5_ 19.53 ± 0.34 μg/mL) used as a standard reference in the only CUPRAC method. In the mentioned antioxidant activity assays, the antioxidant activity of *H. scabrum* essential oils was found to be higher than that of *H. ternatum* samples. As shown in Table 2, the major components in the tested *Hypericum* essential oil samples were found to be similar.

The toxic effects of the tested samples on the healthy cell line (PDF), and their cytotoxic potentials on MCF-7 (breast cancer cell line) and HT-29 (colon cancer cell line) cell lines were determined by the MTT method (Table 2). While none of *H. scabrum* samples showed toxic and cytotoxic effects on PDF and MCF cell lines, respectively, the samples S-1 and S-2 exhibited significant and similar cytotoxic activity in colon cancer cell lines (HT-29). *H. ternatum* samples did not indicate any cytotoxic effect on MCF-7 and HT-29 cell lines, while showing moderate toxic effect on PDF cell line (IC_50_ 35.11 ± 1.32 to 46.93 ± 1.20 μg/mL). Our results exhibited that *H. scabrum* samples collected from different localities had similar essential oil contents as well as their antioxidant, toxic, and cytotoxic activities, like *H. ternatum* samples.

A literature survey indicated that *H. scabrum* ethanol and water extracts exhibited a strong antioxidant effect in DPPH free radical scavenging and β-carotene linoleic acid test systems, and *H. scabrum* essential oils showed moderate activity [8,16]. Keser et al. [16] reported that *H. scabrum* ethanol and water extracts exhibited strong cytotoxic effects on MCF-7, HCT-116, and LNCaP cancer cell lines. This is the first study on the antioxidant activity measured by ABTS cation radical scavenging and CUPRAC methods with the toxic-cytotoxic potentials of *H. scabrum* essential oils. In addition, the antioxidant, toxic-cytotoxic capacities of *H. ternatum* essential oil were determined for the first time in the present work.

### 3.3. Enzyme inhibitory activities

This is the first investigationof the anticholinesterase capacity of *H. ternatum* and *H. scabrum* essential oils. Anticholinesterase activities of the samples were determined based on the inhibition of the acetyl- and butyryl-cholinesterase enzymes (Table 2). All samples primarily S-1 and S-2 (*H. scabrum* samples) (acetylcholinesterase inhibition% 90.33 *±* 1.44 and 93.08 *±* 1.04, and butyrylcholinesterase inhibition% 93.28 *±* 1.99 and 91.08 *±* 2.51, respectively) showed higher anticholinesterase capacity than galantamine (Inhibition% 81.45 ± 0.84 and 78.92 ± 0.65, respectively) used as a standard. *H. ternatum* samples were found to have moderate anticholinesterase activity. The acetyl- and butyryl-cholinesterase inhibitory activity of α-pinene, β-pinene, limonene, and 2-methyloctane were found to be lower than those of the tested essential oils samples. On the other hand, S1–4 essential oils showed higher acetyl- and butyryl-cholinesterase inhibitory activity than galantamine. The significant anticholinesterase potential of S1–4 essential oils could be related to the synergic effect of their constituents.

To the best of our knowledge, there is only one investigation on the anticholinesterase effect of *Hypericum* essential oil. The mentioned work which was carried out by Akdeniz et al. [6] indicated that *H. lysimachioides* var. *spathulatum* essential oil possessed low anticholinesterase activity. In another study, the effects of *H. scabrum* essential oil on anxiety and depressive-like behavior have been assessed in animal models with dementia, which demonstrated that *H. scabrum* essential oil had a neuropharmacological potential [4].

The urease and tyrosinase inhibitory effects of the tested samples are given in Table 2. None of the samples showed urease inhibitory activity. All samples of *H. scabrum*, especially S-1 (Inhibition% 91.66 ± 1.06) and S-2 (Inhibition% 95.25 ± 1.42) samples possessed very high inhibition against tyrosinase. In addition, *H. ternatum* samples exhibited similar moderate tyrosinase inhibitory activity among themselves. To the best of our knowledge, there are a limited number of studies in the literature regarding the tyrosinase inhibitory activity of the essential oils of other *Hypericum* species [6].

The elastase inhibitory capacity of all samples, especially the samples S-1 (Inhibition% 34.59 ± 0.42) and S-2 (Inhibition% 36.69 ± 0.18) of *H. scabrum*, were also found to be high. Moreover, moderate elastase inhibitory activity was also observed for *H. ternatum* samples. All samples were found to have moderate collagenase inhibitory activity (Table 2). To the best of our knowledge, this is the first study on the elastase and collagenase inhibitory potential of *Hypericum* essential oils. However, it is known that the extracts of *Hypericum* species such as olive oil, hexane, ethanol, methanol, and water extracts have high wound healing, antityrosinase, antielastase, and anticollagenase potentials [3].

### 3.4. In vitro and in silico studies of major components

When the in vitro enzyme inhibition activity results of α-pinene, β-pinene, limonene, and 2-methyloctane at 100 μg/ mL concentration were examined, it was determined that they showed low-moderate activity. It was determined that 2-methyloctane and α-pinene compounds, which are the main major component in the samples, showed moderate AChE (Inhibition% 52.47 ± 0.65 and 49.64 ± 0.97, respectively) and antiurease (Inhibition% 43.91 ± 0.18 and 38.76 ± 0.85, respectively) enzyme activities.

**Figure 2:**
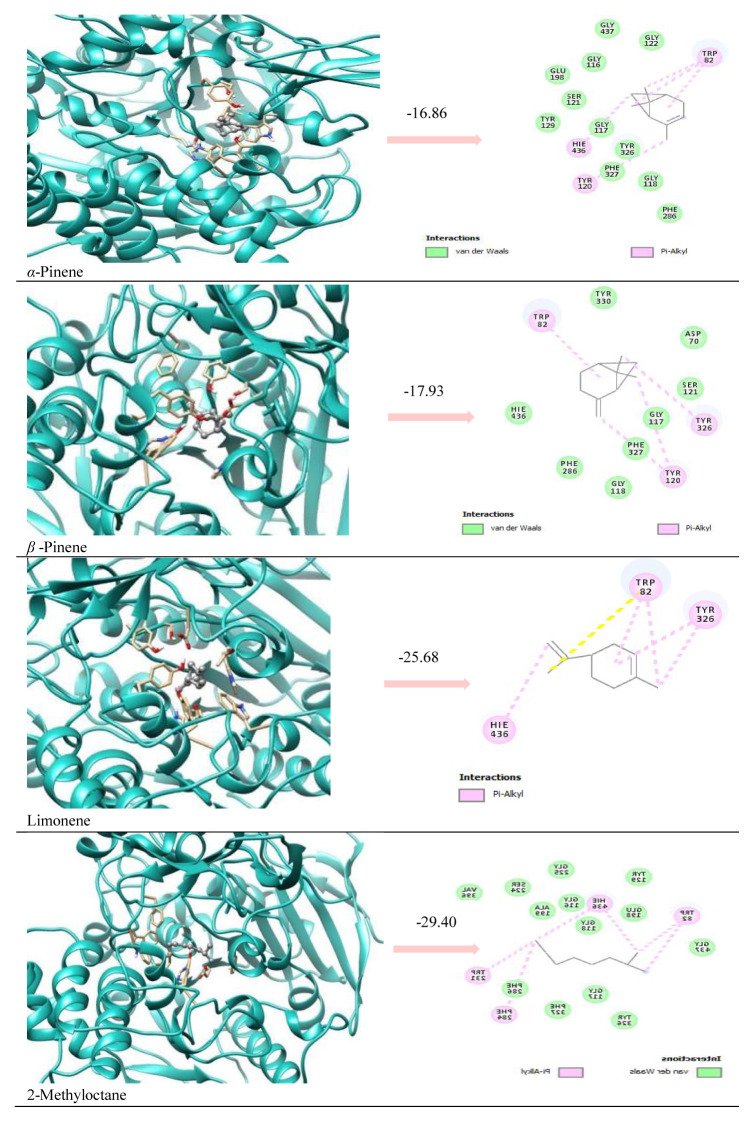
Ribbon representation of the active site pocket enzymes with the bound ligands. The wide opening of the binding site pocket allows the compounds to adopt flexible conformation in this area for AChE.

**Figure 3:**
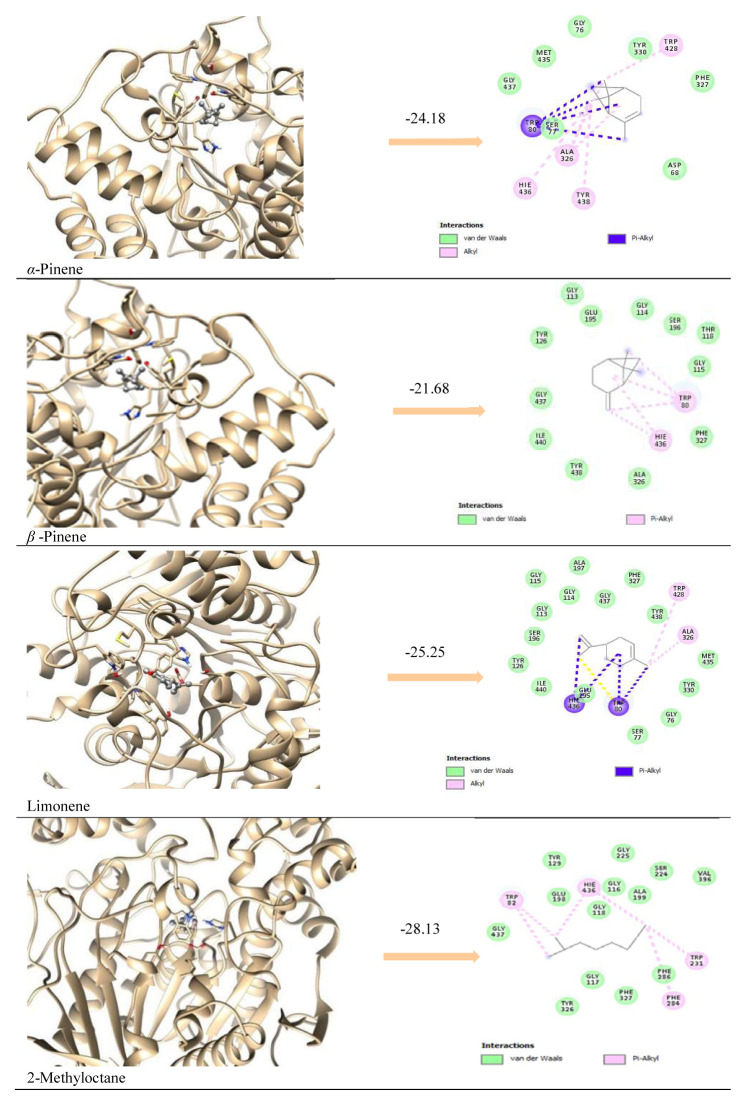
Ribbon representation of the active site pocket enzymes with the bound ligands. The wide opening of the binding site pocket allows the compounds to adopt flexible conformation in this area for BChE.

**Figure 4:**
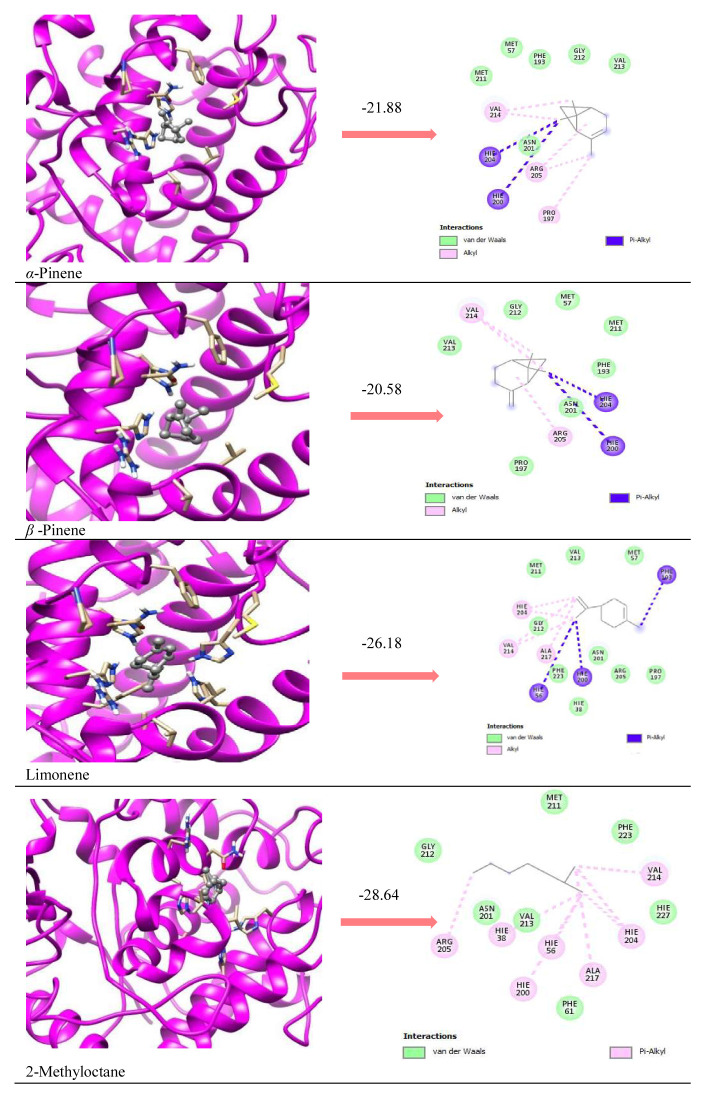
Ribbon representation of the active site pocket enzymes with the bound ligands. The wide opening of the binding site pocket allows the compounds to adopt flexible conformation in this area for tyrosinase.

According to in silico results, enzyme-inhibitor interactions were assessed with docking calculations where binding free energy was recorded at each possible position. Molecular docking result of AChE complexation energy observed in the range from −16.86 kcal/mol to −29.40 kcal/mol, the result of BChE complexation energy observed in the range from −24.18 kcal/mol to −28.14 kcal/mol, the result of tyrosinase complexation energy observed in the range from −21.88 kcal/ mol to −28.64 kcal/mol, the result of elastase complexation energy observed in the range from −17.07 kcal/mol to −25.40 kcal/mol, the result of collagenase complexation energy observed in the range from −22.35 kcal/mol to −28.62 kcal/mol, respectively (Table 3). Since the major components have similar chemical structures, it was determined that they showed activity with similar interactions in all the enzymes studied, with Van der Waals and pi alkyl interactions being dominant (Figures 2–4; Figures S1 and S2).

The in vitro test results of the components identified as major in the essential oils of the species show parallelism with the in silico results. It was determined that the enzyme activities of these components alone were low according to both in vitro and in silico test results. However, it is seen that the anticholinesterase and antityrosinase enzyme activities of the essential oil samples, in which these major components are mixed in certain proportions, are quite high. Likewise, in other enzyme activity methods, it is seen that essential oils are more active than the individual major components. It can be said that the high enzyme activity of essential oils is due to the synergistic effect of the components.

It was determined that the interactions between the prominent natural components and the reference molecules used in the study and their positions in the active site of the enzyme were partially similar. It was observed that reference molecules and ligands made intense vdw and pi-alkyl interactions as seen in Table 4 and Figures 2–4.

The interactions of the active molecules in experimental studies have been clarified by modeling studies. It is compatible with their chemical structures and interaction types and in parallel with the results of in vitro studies.

It is predicted that an attractive and noncovalent interaction between the ligands and the residues Hie, Trp, Tyr, and Phe, which are common in the catalytic region of the AChE and BChE enzymes, plays an important role in the stabilization of the inhibitor in the active site. It is thought that the presence of donor oxygen and nitrogen atoms of the Galantamine molecule, which is used as a reference, differs from the natural components, making a difference in its effectiveness.

If the active binding site of the tyrosinase enzyme is examined, strong interactions are observed with the amino acids Asn, Phe, Ser, and Val, the −OH groups of the reference kojic acid molecule have Vdw and π-π interactions, as well as the specific hydrogen bonds with Met amino acid residues in the active site.

Considering the inhibition effects of elastase enzyme and ligands, some amino acids, such as Asn, Ser, Gly, Cys, and His, play a role in the formation of electrostatic interactions and hydrogen bonds between the ligand and the active amino acids of the protein. Olenaoic acid has the highest affinity with its carboxylic acid functional group, unlike natural components, because it has been determined that it has the highest number of hydrogen bonds to active amino acids, especially Ser and Hie.

Collagenase enzyme active site mainly consists of amino acid residues Gly, Leu, Ala, Glu, Tyr and Pro, while H-bond interactions generally occur with Leu, Ala, Glu, His. As indicated in Table 4, epicatechin gallate used as a reference has a large number of −OH groups and donor oxygen atoms, unlike the natural components showing activity. It is thought that the difference in activity on the enzyme is due to these functional groups (Table 4).

### 3.5. Antimicrobial activity

The tested essential oil samples exhibited relatively close antimicrobial activity to each other. Their MIC values range from 40 to 200 μg/mL. The major effectiveness (MIC value 40 μg/mL) was recorded by S-6 against *S. pyogenes* and *C. albicans*. There are many studies in the literature on the antimicrobial activity of many *Hypericum* species including *H. scabrum*. However, there is no report on the antimicrobial activity of *H. ternatum* (Table 5).

The findings of the current study are compatible with the literature in terms of the high activity of *Hypericum* species. Reports reveal the antimicrobial potential of *H. scabrum* ondifferentmicroorganisms: *Bacillus cereus*, *Listeria monocytogenes*, *Pseudomonas aeruginosa*, and *Salmonella typhimurium* [8]; *Escherichia coli, Proteus vulgaris*, *Klebsiella pneumonia*, *B. subtilis*, *B. megaterium*, *Staphylococcus aureus*, and *Candida albicans* [16]; *Enterococcus faecalis*, *Staphylococcus epidermidis*, *Saccharomyces cerevisiae*, and *Aspergillus niger* [44]; *Brevibacillus brevis* and *Clostridium perfringens* [45].

Heshmati et al. [46] reported moderate antimicrobial activity of *H. scabrum* essential oil and extracts associated with their phenolic content (α-pinene, 3-methyl-β-pinene and β-caryophyllene). Pirbalouti et al. [8] determined variations of antibacterial activity and thymol and carvacrol content in different populations of *H. scabrum* flowers. Compounds with lipophilic character pass more easily through the cell membrane than other molecules. In this respect, terpenoids and their derivatives can easily pass through the cell membrane of microorganisms and adversely affect the membrane structure, ion transport, and cellular respiration. The high antimicrobial activities of *Hypericum* species could be explained with the presence of α-pinene, β-pinene, and (*E*)-caryophyllene known for their antimicrobial potential [47].

## 4. Conclusion

Although *Hypericum* species have attracted extensive interest mainly due to their antidepressant effects, it is known that many species belonging to the genus *Hypericum*, especially *H. perforatum*, have been used for topical applications in the treatment of dermatological diseases since ancient times [3]. A literature survey showed that the tyrosinase, collagenase, elastase, and hyaluronidase inhibitory capacities of many *Hypericum* species, especially *H. perforatum*, with their antiinflammatory and wound healer effects were investigated, and the reported results possessed an important value for cosmetic industry.

In this regard, the essential oil contents of *H. scabrum* and *H. ternatum* collected from different localities in Turkey were determined by GC-MS/FID. Furthermore, their antimicrobial, cytotoxic, and antioxidant potentials as well as their anticholinesterase, antiurease, antityrosinase, antielastase, and anticollagenase effects were also defined.

GC-MS/FID results indicated that the essential oils obtained from the plant materials collected from different localities possessed similar chemical contents: the major components of *H. scabrum* samples (S-1, S-2, S-3, and S-4) were α-pinene (56.25, 62.80, 58.61 and 55.96%, respectively), while the main constituents of *H. ternatum* samples (S-5, S-6, and S-7) were found to be 2-methyloctane (18.67, 22.39 and 9.45%, respectively) and α-pinene (16.45, 33.08 and 12.79%, respectively), and the biological tests exhibited that they have similar activity capacity. *H. scabrum* essential oils showed much higher biological activity potential than those of *H. ternatum* samples, except for the antimicrobial activity.

It has been determined that the essential oil samples of the *H. scabrum* species usually have a high potential especially in terms of anticholinesterase, tyrosinase, collagenase, and elastase enzyme activities. When the *H. perferatum* species in the literature is compared with these enzyme studies, it can be said that *H. scabrum* has a comparable potential. As a result, our findings suggest that *H. scabrum* essential oil could be an alternative source to *H. perforatum* in the cosmetic and pharmaceutical industries to treat dermatological disorders, memory loss, colon cancer, etc. Moreover, it is thought that essential oils show high enzyme activities due to the synergistic effect arising from their coexistence, not from their main components alone.

## SUPPLEMENTARY MATERIAL

Figure S1 Ribbon representation of the active site pocket enzymes with the bound ligands. The wide opening of the binding site pocket allows the compounds to adopt flexible conformation in this area for elastase.

Figure S2 Ribbon representation of the active site pocket enzymes with the bound ligands. The wide opening of the binding site pocket allows the compounds to adopt flexible conformation in this area for collagenase.

## Figures and Tables

**Figure 1A:**
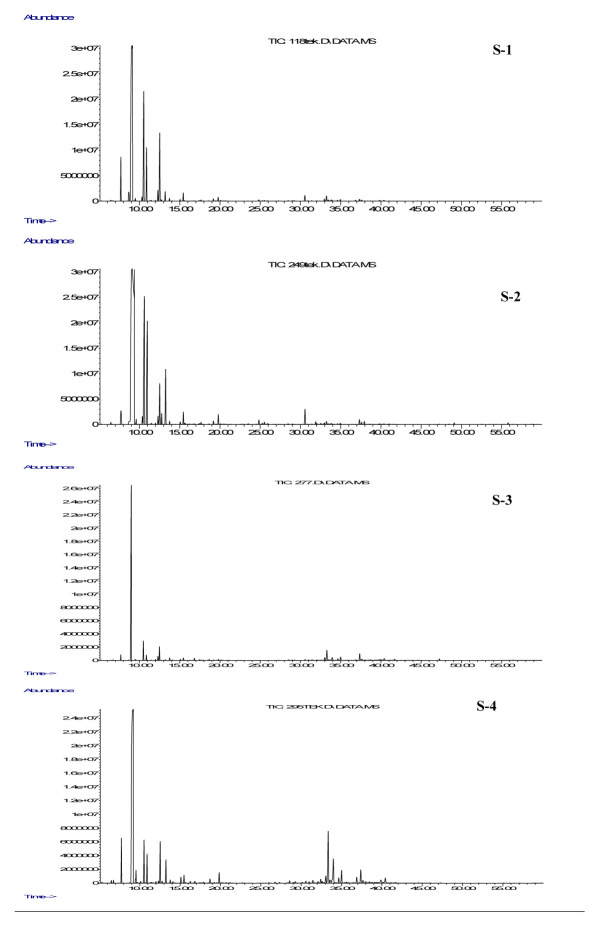
Total ion chromatogram (TIC) of *Hypericum scabrum* essential oils by GC-MS. **S-1:** *H. scabrum* collected in Nevşehir, **S-2:** *H. scabrum* collected in Diyarbakır, **S-3:** *H. scabrum*, collected in Kahramanmaraş, S-4: H. scabrum collected in Van.

**Figure 1B:**
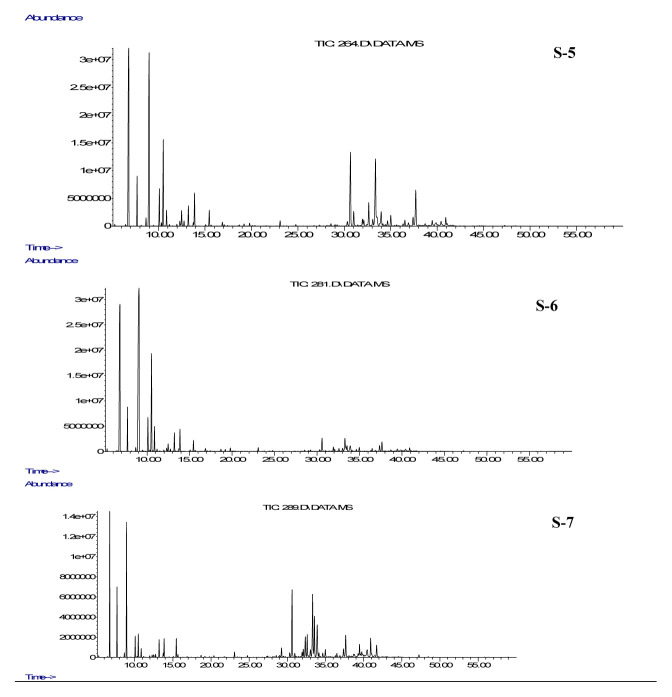
Total ion chromatogram (TIC) of *Hypericum ternatum* essential oils by GC-MS. **S-5:** *H. ternatum* collected in Amasya, **S-6:** *H. ternatum* collected in Tekirdağ, **S-7:** *H. ternatum* collected in Afyonkarahisar.

**Table 1:** Essential oil compositions of *Hypericum scabrum* and *H. ternatum* samples.

No	RI^a^	Constituents^b^	S-1^c^	S-2	S-3	S-4	S-5	S-6	S-7	No	RI^a^	Constituents^b^	S-1^c^	S-2	S-3	S-4	S-5	S-6	S-7
1	848	2-Hexenal	0.12	0.13	0.12	0.21	0.12	0.11	0.13	36	1427	Caryophyllene	0.77	1.19	0.41	0.30	8.35	1.78	9.50
2	859	2-Methyloctane	0.14	0.15	0.13	0.20	18.67	22.39	9.45	37	1446	Aromadendrene	-	-	0.30	0.33	0.12	-	-
3	899	Nonane	3.70	1.34	1.17	3.17	2.67	3.12	4.67	38	1457	α-Himachalene	-	0.20	-	-	-	-	-
4	927	α-Thujene	1.54	-	0.12	-	0.52	0.38	tr	39	1458	*trans*-β-Farnesene	-	-	-	-	-	-	0.61
5	935	α-Pinene	56.25	62.80	58.61	55.96	16.45	33.08	12.79	40	1460	*cis*-β-Famesene	-	-	-	-	0.69	0.52	-
6	950	Camphene	0.29	0.32	0.34	1.14	0.12	0.16	0.14	41	1462	Humulene	0.10	0.13	0.26	0.20	0.62	0.26	0.97
7	967	Nonane, 3-methyl	0.20	0.12	0.11	0.12	2.46	3.03	1.60	42	1473	Cyclodecane	-	-	-	0.22	2.22	0.39	2,64
8	975	Sabinene	0.45	0.54	-	-	-	-	0.10	43	1483	γ-Muurolene	0.12	0.22	1.23	1.02	0.93	0.50	1.34
9	981	β-Pinene	13.67	11.25	5.11	3.37	6.24	10.22	1.94	44	1490	Germacrene D	0.72	0.28	4.10	6.42	8.63	1.96	9.36
10	991	β-Myrcene	5.18	8.06	1.38	2.22	1.04	2.12	0.75	45	1495	β-Selinene	-	-	0.37	0.36	1.13	0.84	5.04
11	999	Decane	-	-	-	-	0.11	0.21	-	46	1501	Valencene	-	-	0.31	-	0.31	-	-
12	1018	α-Terpinene	-	-	0.28	0.13	-	0.15	0.14	47	1503	α-Selinene	-	-	-	-	-	1.26	5.41
13	1026	*p*-Cymene	1.13	0.49	1.18	0.18	0.44	0.40	0.24	48	1505	Bicyclogermacrene	-	-	1.56	3.69	1.80	-	-
14	1030	Limonene	8.46	2.73	3.89	3.61	1.24	0.81	0.37	49	1509	α-Farnesene	-	-	-		0.23	0.15	0.53
15	1036	trans-β-Ocimene	0.13	0.60	0.12	0.16	0.36	0.23	0.27	50	1521	γ-Cadinene	0.14	0.07	0.67	0.61	1.74	0.83	1.05
16	1047	*cis*-β-Ocimene	0.91	3.53	0.21	1.90	1.42	1.70	1.55	51	1530	δ-Cadinene	0.25	0.10	1.59	1.54	0.12	0.10	1.16
17	1059	γ-Terpinene	0.29	0.19	0.71	0.29	0.27	0.27	0.15	52	1551	α-Calacorene	-	-	0.17	-	0.11	-	-
18	1062	Decane, 2-methyl	-	-	-	-	2.40	2.02	1.63	53	1566	Nerolidol	-	-	-	-	0.59	0.32	0.41
19	1091	Terpinolene	0.20	0.17	0.50	0.60	-	0.19	-	54	1587	Spathulenol	0.27	0.40	2.67	1.62	1.05	0.78	1.19
20	1099	Undecane	0.83	0.76	0.79	0.70	1.16	1.03	1.75	55	1594	Caryophyllene oxide	0.10	0.21	0.62	0.41	4.41	1.42	3.18
21	1104	Nonanal	-	-	-	0.11	0.10	0.13	0.28	56	1602	Viridiflorol	-	0.27	-	0.29	0.17	-	-
22	1169	*endo*-Borneol	-	-	0.34	0.44	-	-	-	57	1613	Globulol	0.19	-	-	0.25	-	-	2.15
23	1180	Terpinene-4-ol	0.28	0.25	0.23	0.12	0.19	0.25	tr	58	1620	Bisabolene epoxide	-	-	-	-	0.31	-	0.48
24	1193	α-Terpineol	0.46	0.68	0.39	-	0.26	0.39	-	59	1630	Junenol	-	-	-	-	0.12	-	-
25	1200	Myrtenol	0.18	-	0.25	-	0.11	-	-	60	1640	1-Dodecanol	-	-	-	-	0.72	-	1.57
26	1263	Dodecane, 2-methyl	-	-	-	-	0.48	0.44	0.57	61	1649	*tau*-Cadinol	0.15	tr	0.67	0.63	0.75	0.37	0.41
27	1299	Tridecane	-	-	-	-	0.14	0.12	0.18	62	1663	α-Cadinol	0.14	-	1.32	0.83	0.79	0.59	tr
28	1354	α-Cubebene	0.04	-	-	0.12	-	-	-	63	1676	1-Tetradecene	-	-	-	-	0.85	0.48	2.40
29	1358	α-Longipinene	-	-	-	-	-	-	0.17	64	1846	Hexahydrofarnesyl acetone	-	-	0.60	-	-	-	-
30	1370	β-Cubebene	-	-	0.25	0.16	-	0.10	-		Total identified (%)		97.70	97.4	93.51	94.33	94.69	96.11	90.09
31	1376	α-Ylangene	0.02	-	-	0.11	-	-	-		Monoterpene hydrocarbons		88.7	90.8	72.9	70.34	32.89	53.34	22.68
32	1382	α-Copaene	0.11	0.11	0.26	0.26	0.23	0.16	0.21		Oxygenated monoterpenes		0.92	0.93	0.87	0.12	0.56	0.64	0.00
33	1391	β-Bourbonene	0.10	0.11	0.17	0.12	0.20	0.15	0.42		Sesquiterpene hydrocarbons		2.45	2.41	11.65	15.45	25.74	8.81	36.96
34	1396	β-Elemene	0.08	-	-	0.21	-	0.20	1.19		Oxygenated sesquiterpenes		0.85	0.88	5.28	4.03	8.19	3.48	7.82
35	1422	α-Cedrene	-	-	-	-	0.53	-	-		Others		4.79	2.38	2.81	4.39	27.31	29.84	22.63

a Retention index on HP–5MS fused silica column,

b A nonpolar Agilent HP-5MS fused silica column,

c Percentage concentration, **S-1:** *H. scabrum* collected in Nevşehir, **S-2:** *H. scabrum* collected in Diyarbakır, **S-3:** *H. scabrum* collected in Kahramanmaraş, **S-4:** *H. scabrum* collected in Van, **S-5:** *H. ternatum* collected in Amasya, **S-6:** *H. ternatum* collected in Tekirdağ, **S-7:** *H. ternatum* collected in Afyonkarahisar.

**Table 2:** Biological activities of *Hypericum scabrum* and *H. ternatum* essential oils^1^.

	Antioxidant activity			Cytotoxic activity			Enzyme activity (100 μg/mL)					
	IC_50_ (μg/mL)		A_0.5_ (μg/mL)	IC_50_ (μg/mL)			Inhibition (%)					
Samples^2^	DPPH	ABTS	CUPRAC	HT-29	MCF-7	PDF	AChE	BChE	Urease	Tyrosinase	Elastase	Collagenase
S-1	> 250	38.04 ± 0.65^a^	7.95 ± 0.02^a^	21.67 ± 0.34^a^	111.79 ±1.19^a^	78.43 ± 0.49^a^	90.33 ± 1.44^a^	93.28 ± 1.99^a^	NA^a^	91.66 ± 1.06^a^	34.59 **±** 0.42^a^	27.53 **±** 0.43^a^
S-2	> 250	67.07 ± 0.28^b^	10.60 ± 0.65^b^	24.57 ± 1.04^b^	102.52 ± 0.87^a^	87.09 ± 1.12^b^	93.08 ± 1.04^b^	91.08 ± 2.51^b^	NA^a^	95.25 ± 1.42^b^	36.69 **±** 0.18^b^	31.70 **±** 0.58^b^
S-3	> 250	198.91 ± 0.92^c^	134.96 ± 5.28^c^	34.67 **±** 045^c^	98.09 **±** 1.03b	79.48 **±** 0.59^a^	83.30 ± 1.90^c^	80.58 ± 1.19^c^	NA^a^	70.57 ± 1.53^c^	27.88 **±** 0.34^c^	14.80 **±** 0.13^c^
S-4	> 250	179.21 ± 0.78^d^	> 250^d^	31.67 **±** 0.75^d^	103.09 **±** 1.41^a^	97.05 **±** 0.59^b^	89.08 ± 1.17^a^	88.15 ± 2.95^d^	NA^a^	69.00 ± 1.64^c^	28.50 **±** 0.03^c^	15.57 **±** 0.06^c^
S-5	> 250	184.30 ± 1.03^e^	> 250^d^	89.69 ± 0.65^e^	76.23 ± 1.03^c^	43.19 ± 0.98^c^	38.47 ± 1.26^d^	61.99 ± 2.34^e^	NA^a^	31.34 ± 1.47^d^	14.00 **±** 0.01^d^	10.99 **±** 0.11^d^
S-6	> 250	> 250^f^	> 250^d^	109.89 **±** 1.42^f^	231.83 **±** 1.59^d^	35.11 ± 1.32^d^	67.67 ± 1.06^e^	57.22 ± 0.80^f^	NA^a^	54.75 ± 1.48^e^	14.50 **±** 0.13^d^	12.47 **±** 0.12^e^
S-7	> 250	> 250^f^	177.94 ± 3.56^e^	94.59 ± 0.18^g^	111.02 ± 1.89^a^	46.93 ± 1.20^e^	40.62 ± 2.74^f^	72.83 ± 1.58^g^	NA^a^	31.13 ± 1.52^f^	16.02 **±** 0.21^e^	11.51 **±** 0.15^d^
α-Pinene	-	-	-	-	-	-	49.64 ± 0.97^g^	11.93 ± 0.04^h^	38.76 ± 0.85^b^	NA^g^	16.31 **±** 0.11^f^	12.86 **±** 0.10^e^
β-Pinene	-	-	-	-	-	-	NA^h^	NA^i^	19.58 ± 0.45^c^	NA^g^	21.80 ± 0.45^g^	9.54 **±** 0.02^f^
Limonene	-	-	-	-	-	-	NA^h^	9.22 ± 0.02^j^	8.29 ± 0.05^d^	NA^g^	17.79 ± 0.23^f^	NA^g^
2-Methyloctane	-	-	-	-	-	-	52.47 ± 0.65^i^	17.41 ± 0.62^k^	43.91 ± 0.18^e^	12.03 ± 0.21^h^	24.20 **±** 0.06^h^	16.59 **±** 0.81^h^
BHT^3^	54.68 **±** 0.47^a^	15.24 **±** 0.63^g^	8.42 ± 0.25^a^	-	-	-	-	-	-	-	-	-
α-TOC^3^	14.55 **±** 0.26^b^	9.52 **±** 0.36^h^	19.53 ± 0.34^f^	-	-	-	-	-	-	-	-	-
Galantamine^3^	-	-	-	-	-	-	81.45 **±** 1.84^j^	78.92 **±** 0.65^l^	-	-	-	-
Thiourea^3^	-	-	-	-	-	-	-	-	96.75 ± 0.42^f^	-	-	-
Kojic acid^3^	-	-	-	-	-	-	-	-	-	87.73 ± 0.48^i^	**-**	**-**
Oleanolic acid^3^	-	-	-	-	-	-	-	-	-	-	42.61 ± 0.28^i^	-
Epicatechin gallate^3^	-	-	-	-	-	-	-	-	-	-	-	83.84 ± 1.78^i^

1 Values expressed are means ± SD of three parallel measurements and values were calculated according to negative control. Values with different letters in the same column were significantly different (p < 0.05)

2 **S-1:** *H. scabrum* collected in Nevşehir, **S-2:** *H. scabrum* collected in Diyarbakır, **S-3:** *H. scabrum* collected in Kahramanmaraş, **S-4:** *H. scabrum* collected in Van, **S-5:** *H. ternatum* collected in Amasya, **S-6:** *H. ternatum* collected in Tekirdag, **S-7:** *H. ternatum* collected in Afyonkarahisar.

3 Standard compound, NA: Not active

**Table 3:** Calculated thermodynamic parameters for complexation of ligands by docking method.

Compounds	AChE			BChE			Tyrosinase			Elastase			Collagenase		
	VdW	es	DockS	VdW	es	DockS	VdW	es	DockS	VdW	es	DockS	VdW	es	DockS
α-Pinene	−16.77	−0.093	−16.86	−23.998	−0.18	−24.18	−21.85	−0.03	−21.88	−16.95	−0.12	−17.07	−22.16	−0.182	−22.35
β-Pinene	−17.91	−.0.013	−17.93	−21.66	−0.02	−21.68	−20.50	−0.08	−20.58	−16.17	−0.07	−16.24	−23.01	−0.46	−23.46
Limonene	−25.44	−0.25	−25.68	−25.13	−0.12	−25.25	−25.98	−0.21	−26.18	−24.14	−0.09	−24.05	−26.72	−0.11	−26.72
2-Methyloctane	−29.37	−0.030	−29.40	−0.004	−28.13	−28.14	−0.059	−28.58	−28.64	−0.11	−25.29	−25.40	−0.074	−28.55	−28.62
Galantamine	−66.53	−11.68	−78.21	−63.05	−7.14	−71.19	-	-	-	-	-	-	-	-	-
Kojic Acid	-	-	-	-	-	-	−69.19	−10.97	−80.16	-	-	-	-	-	-
Oleanolic acid	-	-	-	-	-	-	-	-	-	−31.39	−0.86	−32.25	-	-	-
Epicatechin gallate	-	-	-	-	-	-	-	-	-	-	-	-	−7.20	−53.90	−61.11

VdW: Van der Waals energy, es: electrostatic energy, DockS: Dock score energy, all energy parameters in kcal/mol

**Table 4:** Interaction of compounds in the binding pocket of enzymes.

Kod	AChE			BChE			Tyrosinase			Elastase			Collagenase		
	VdW	Pi-alkyl	Alkyl	VdW	Pi-alkyl	Alkyl	VdW	Pi-alkyl	Alkyl	VdW	Pi-alkyl	Alkyl	VdW	Pi-alkyl	Alkyl
α-Pinene	Gly437 Gly122 Tyr326 Phe327	Hie436, Tyr320	-	Gly437 Met435 Gly 78Asp 68	Trp 80	Trp428 Ala326 Tyr438 Hie436	Met 57 Gly212 Phe193 Asn201	Hie204 Hie200	Val214 Arg205 Pro197	Leu88 Phe209 Gly210	-	Hie42	Gly80 Hie129 Pro139 Tyr139	Hie119	Leu82
β-Pinene	Trp 82 Gly1182Gly 117 Tyr326 Phe 286Phe 206	Hie436 Trp82 Tyr122	-	Gly113 Gly114 Ile437 Ala326 Tyr438 Ser196	Trp80, Hie436	-	Gly212 Met57 Met211 Val213 Pro197 Asn201	Hie204 Hie200	Val214 Arg205	Leu88 Asn182 Ser188 Gly210 Phe209	-	Hie42			
Limonene	-	-	Trp82 Tyr326 Hie436	Gly113 Gly115 Phe327 Tyr438 Ser196 Tyr126 Ile440	-	Hie436 Trp80	Val213 Met211 Met57 Gly212 Asn201 Phe223	Hie56 Hie200	Ala217 Val214 Hie204 Ala217	Asp187 Se183Cys184 Asn185 Gly214	-	Val207 Cys215	Leu136 Tyr141 Ile140 Pro139 Ala83 Hi3123	Hie119	Val116 Leu81 Leu82
2-Methyloctane	Tyr129 Glu225 Ser224 Val396 Gly117 Phe286 Phe327 Gly118	-	Trp82 Hie436 Trp231 Phe284	Tyr129 Gly225 Ser224 Val396 Ala199 Phe327 Gy117Tyr326	-	Hie436 Trp231 Trp82 Phe284				Gly186 Ser208 Gly210 Asn185 Ser212 Ser183 Cys215 Thr137 Asp187	-	Val207	Leu136 Pro139 Glu120 Ala 83 Hie129 Val116 Ile140	-	Leu115 Tyr141 Hie119
Galantamine	Gly1182Gly117 Tyr326	Gly118 Trp82 Tyr122	Phe286 Phe327												
Kojic acid							Gly80 Hie129 Pro139 Tyr139	Hie119	Leu82						
Oleanolic acid										Ser212 Ser183 Cys215 Thr13 Asp187 Gly210	-	Hie 42 Cys215			
Epicatechin gallate													Glu120 Ala 83 Hie129 Val116	Hie119	Leu115 Tyr141 Hie119 Leu82

**Table 5:** Antimicrobial activity of *Hypericum scabrum* and *H. ternatum* essential oils (MIC values as μg/mL).

	Microorganisms				
Samples^a^	Gram-negative		Gram-positive		Yeast
	*E. coli*	*P. aeruginosa*	*S. aureus*	*S. pyogenes*	*C. albicans*
S-1	50 ± 0.5	200 ± 0.2	60 ± 0.3	50 ± 0.7	50 ± 0.7
S-2	70 ± 0.2	200 ± 0.1	60 ± 0.1	50 ± 0.4	50 ± 0.3
S-3	80 ± 0.3	70 ± 0.4	60 ± 0.3	50 ± 0.8	50 ± 0.8
S-4	80 ± 0.6	200 ± 0.1	60 ± 0.1	50 ± 0.4	50 ± 0.3
S-5	50 ± 0.6	90 ± 0.4	90 ± 0.3	50 ± 0.8	50 ± 0.8
S-6	50 ± 0.5	50 ± 0.2	50 ± 0.3	40 ± 0.7	40 ± 0.7
S-7	60 ± 0.3	80 ± 0.4	70 ± 0.3	50 ± 0.8	50 ± 0.8
PC^b^	7.8 ± 0.4	NA	95 ± 0.37	7.81 ± 0.1	3.12 ± 0.2

a **S-1:** *H. scabrum* collected in Nevşehir, **S-2:** *H. scabrum* collected in Diyarbakır, **S-3:** *H. scabrum* collected in Kahramanmaraş, **S-4:** *H. scabrum* collected in Van, **S-5:** *H. ternatum* collected in Amasya, **S-6:** *H. ternatum* collected in Tekirdag, **S-7:** *H. ternatum* collected in Afyonkarahisar.

b PC: positive controls that are ampicillin for bacteria and fluconazole for yeast, NA: not active
