# Supplementary figures and images for: Determination of essential oil and biological activities of Hypericum ternatum Poulter and H. scabrum L. species collected from different localities: is H. scabrum an alternative to multifunctional species ST JOHN’S WORT (H. perforatum)?
Source: Turk J Chem. 2022 Aug 13;46(6):1956–71. doi: 10.55730/1300-0527.3494 (PMC10446943; doi:10.55730/1300-0527.3494)

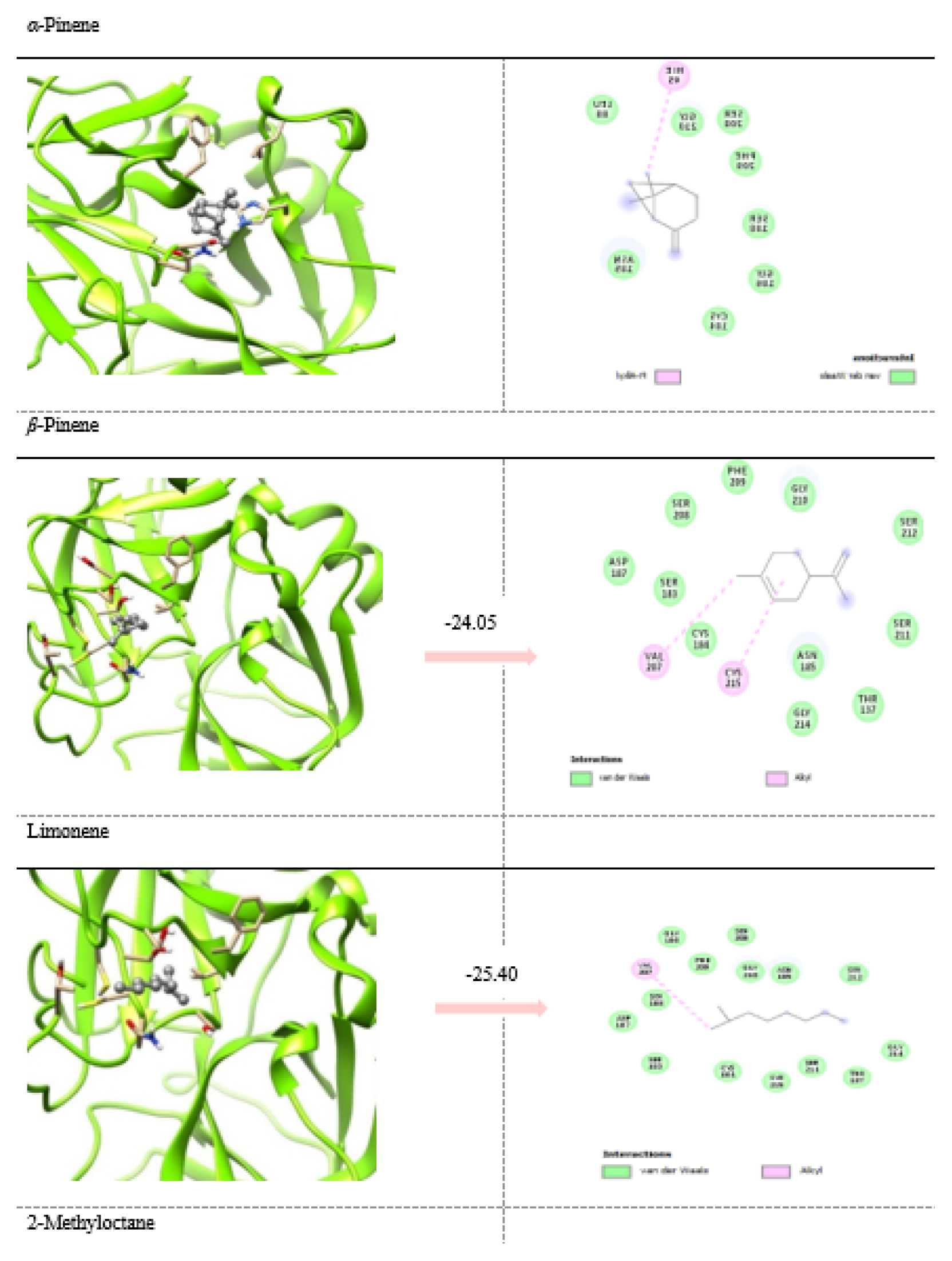

Supplement: Figure S1 — Ribbon representation of the active site pocket enzymes with the bound ligands. The wide opening of the binding site pocket allows the compounds to adopt flexible conformation in this area for elastase. [file turkjchem-46-6-1956s1.tif]

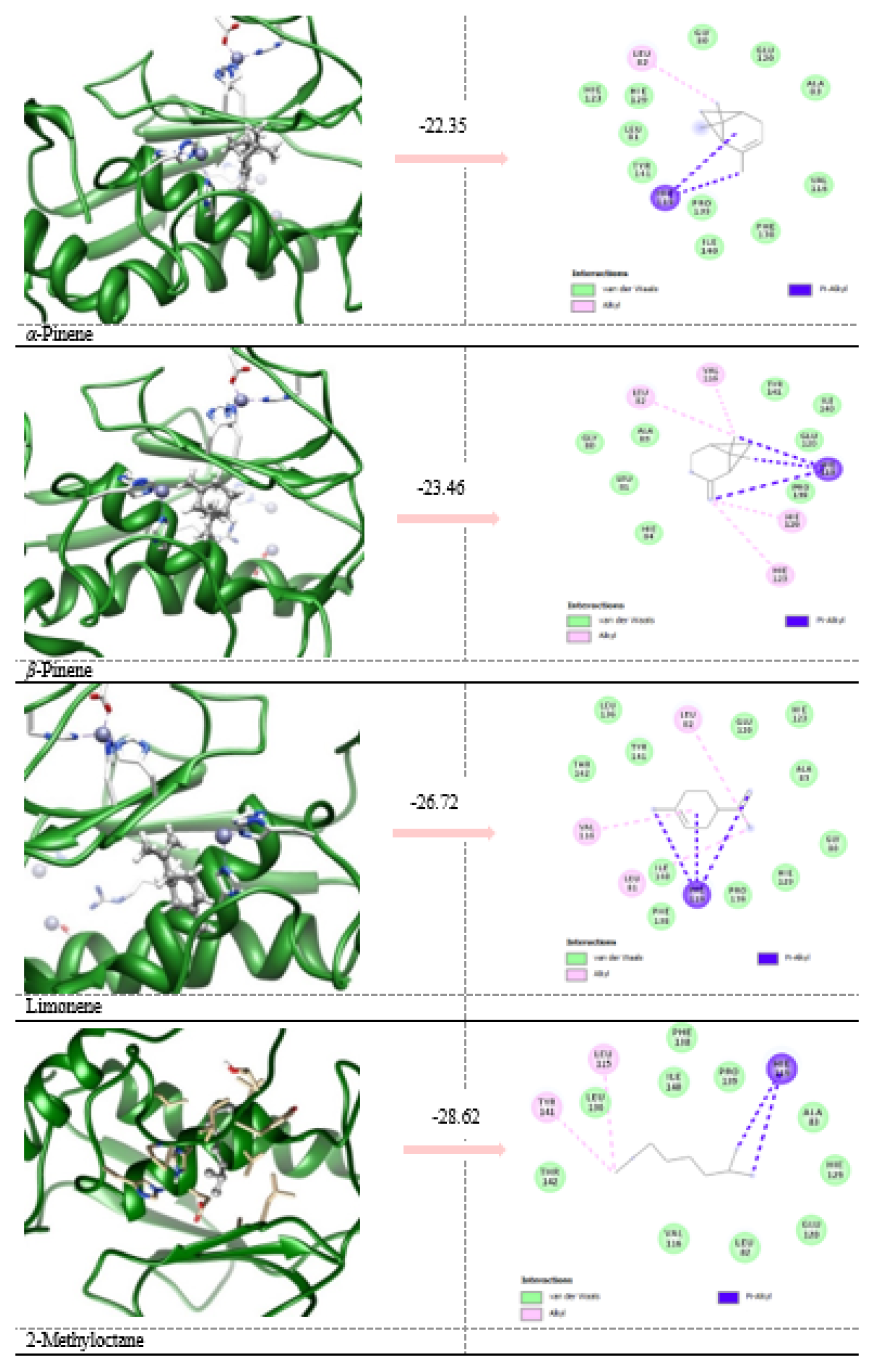

Supplement: Figure S2 — Ribbon representation of the active site pocket enzymes with the bound ligands. The wide opening of the binding site pocket allows the compounds to adopt flexible conformation in this area for collagenase. [file turkjchem-46-6-1956s2.tif]
